# Coordination of network heterogeneity and individual preferences promotes collective fairness

**DOI:** 10.1016/j.patter.2025.101293

**Published:** 2025-06-16

**Authors:** Xiao Han, Shangmei Ma, Wen-Xu Wang, Angel Sánchez, H. Eugene Stanley, Shinan Cao, Boyu Zhang

**Affiliations:** 1 School of Systems Science, Beijing Jiaotong University, Beijing 100044, P.R. China; 2 School of Systems Science, Beijing Normal University, Beijing 100875, P.R. China; 3 Grupo Interdisciplinar de Sistemas Complejos (GISC), Departamento de Matemáticas, Universidad Carlos III de Madrid, Madrid, Spain; 4 Instituto de Biocomputación y Física de Sistemas Complejos (BIFI), Universidad de Zaragoza, 50018 Zaragoza, Spain; 5 Center for Polymer Studies and Department of Physics, Boston University, Boston, MA 02215, USA; 6 School of Finance, University of International Business and Economics, Beijing 100029, P.R. China; 7 School of Mathematical Sciences, Beijing Normal University, Beijing 100875, P.R. China

**Keywords:** coordination, fairness, network heterogeneity, individual preference, ultimatum game

## Abstract

There are intensive debates about whether heterogeneous networks promote prosocial behaviors such as fairness and cooperation. Theoretical models predict that network heterogeneity plays a positive role, but this prediction has not been validated by experiments. We reconcile this debate by conducting experiments with two-stage ultimatum games on networks. In the first stage, we identify responders with strong fairness preferences, referred to as leaders. In the second stage, when leaders occupy high-degree nodes in a heterogeneous network, their ability to motivate fairness among neighboring proposers is amplified, and collective fairness is facilitated. We propose an evolutionary game model and an agent-based simulation framework that capture the microscopic mechanisms underlying the networked experiments. Our experiments, model, and simulations suggest that network reciprocity is achievable but requires coordinated interactions between different prosocial inclinations of individuals and social network structures.

## Highlights

• Network heterogeneity improves fairness when leaders occupy influential nodes
• Leaders pressure selfish proposers to increase offers through rejections
• Ordinary responders show fairness when indirectly influenced by leaders
• Collective fairness arises from network-preference diversity synergy

## The bigger picture

Promoting fairness in communities is essential. While network reciprocity theory suggests that the structure of networks can influence fairness, experimental evidence has been mixed across different societies and contexts. Our study demonstrates that fairness norms emerge not only from individual characteristics but also from an individual’s position within a network. We show that collective fairness can develop in a structured population when a few fairness-driven individuals hold key positions in the network. This insight highlights how social diversity and structural influence interact to shape group behavior. Our findings provide guidance for enhancing fairness in complex systems, where cooperation and equity are crucial yet challenging to enforce.

## Graphical abstract

**Figure:**
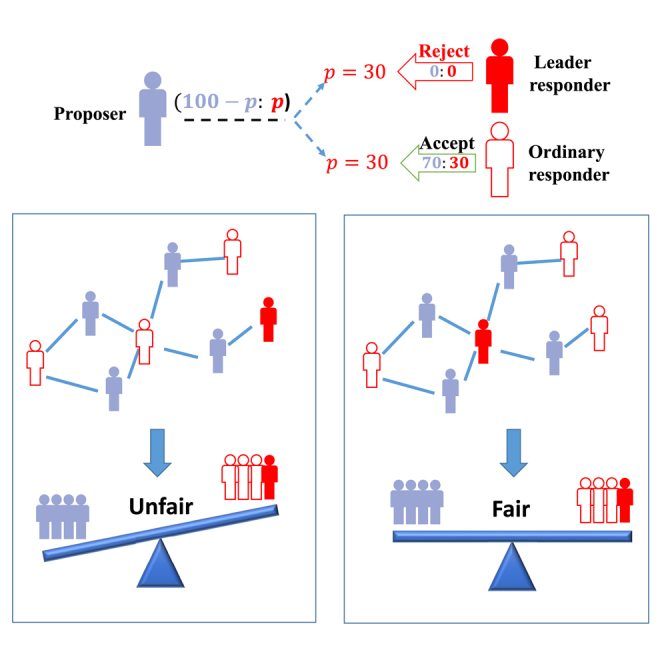

## Teaser

The authors investigate how network structure and preference diversity jointly influence collective fairness using behavioral experiments, an evolutionary game model, and agent-based simulations. They find that while network heterogeneity can promote fairness, this outcome depends on coordinated interactions between individuals with diverse prosocial tendencies and the underlying network topology. In particular, fairness-driven individuals in central network positions can trigger collective fairness, highlighting that network reciprocity emerges not automatically, but through alignment between structural connectivity and behavioral diversity.

## Introduction

Fairness plays a pivotal role in shaping behavior across human and animal societies, as well as in key domains, such as artificial intelligence, healthcare, and climate change.^1^^,^^2^^,^^3^^,^^4^ In fact, without mechanisms that ensure at least a minimal degree of fairness in the distribution of resources and payoffs, cooperation among genetically unrelated individuals cannot be maintained.^5^^,^^6^^,^^7^^,^^8^ This aligns with evidence that the sense of fairness is both cross-cultural in human societies and prevalent among animal species.^9^^,^^10^^,^^11^^,^^12^ On the other hand, human preferences for fairness appear to be more pronounced than those observed in other primates, such as chimpanzees,^13^ in agreement with the fundamental role that fair behavior has played in enabling the large-scale cooperation characteristic of human societies for at least the past 10,000 years.^6^^,^^7^^,^^14^ Moreover, individuals’ fairness preferences are usually relatively stable over time or across different contexts, but they can also evolve due to changing circumstances, such as exposure to different social norms.^15^^,^^16^ Despite its importance, the emergence of human fairness remains an evolutionary puzzle, and the mechanisms that drive the uniquely human inclination toward fairness remain elusive.

One possible explanation for the prevalence of fairness in human society is the presence of large-scale social networks composed of genetically unrelated individuals within human groups. Such networks are rare among animal species and may represent the defining characteristic that distinguishes uniquely fair human behavior. Empirical studies indicate that social networks often exhibit heterogeneous and small-world characteristics,^17^^,^^18^^,^^19^ suggesting that network effects may play a significant role in shaping the norms associated with prosocial behavior.^20^^,^^21^^,^^22^^,^^23^^,^^24^^,^^25^^,^^26^^,^^27^^,^^28^ In the context of this hypothesis, a substantial amount of research on fairness is based on the ultimatum game (UG) and its variants.^29^ In the standard UG, two players—a proposer and a responder—determine how to divide a resource. The proposer suggests a portion of the resource to the responder, who can either accept or reject the offer. If the responder accepts, the resource is allocated according to the proposed terms; if the responder rejects, neither player receives any payoff. The UG has served as a simple yet highly effective theoretical and experimental framework for studying fairness for the past 40 years.^22^^,^^30^^,^^31^^,^^32^^,^^33^^,^^34^^,^^35^^,^^36^^,^^37^^,^^38^ However, although theoretical and numerical results predict that network heterogeneity promotes fair offers and their acceptance in the absence of reputation effects,^21^^,^^23^^,^^39^^,^^40^^,^^41^^,^^42^^,^^43^^,^^44^ this prediction has not been observed in laboratory experiments. On the contrary, some experiments on large-scale networks have found that network heterogeneity has little impact on fair or cooperative behavior.^24^^,^^25^^,^^26^^,^^38^^,^^45^ Despite numerous experimental attempts, only regular networks—which are not accurate representations of society or human communities in general—appear to affect decision-making positively, even then, only under specific conditions.^27^ How network heterogeneity influences fairness remains a perplexing question for scientific communities across a wide range of fields.

In this paper, we advance the understanding of the combined effects of heterogeneous network structures and individuals’ fairness preferences on allocation fairness and efficiency through a two-stage UG laboratory behavioral experiment. In the first stage of our experiment there are no steady social ties, allowing us to identify leader responders with a strong fairness preference. In the second stage, responders and proposers are placed on the nodes of a static bipartite network. Our results show that network heterogeneity can promote allocation fairness, but this ability cannot be fully effective unless the strong fairness-preference leaders are located at influential nodes (i.e., hubs in heterogeneous networks). Under the influence of strong fairness-preference leaders, self-interested proposers are compelled to increase their offers to avoid punishment from the leaders in the form of offer rejection. Furthermore, ordinary responders (i.e., those who do not exhibit strong fairness preferences) display conditional fairness behavior, indirectly motivated by the leaders. Ultimately, high levels of allocation fairness are achieved both collectively and individually, leading to greater allocation efficiency. As we discuss below, we find that fairness within populations is promoted by social diversity, specifically through the combined effect of network heterogeneity and distinct fairness preferences. Neither network heterogeneity nor individual diversity alone is sufficient to support a high level of collective allocation fairness and efficiency.

## Results

### The laboratory behavioral experiment

**Figure 1:**
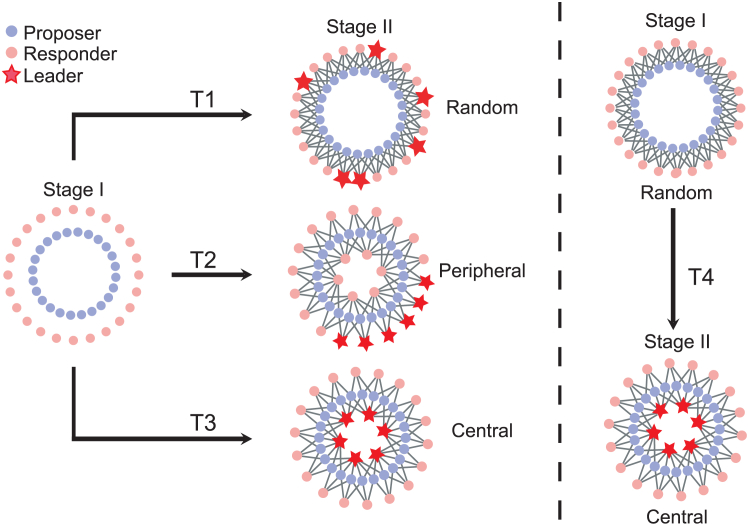
Schematic illustration of the experimental design The experiment consists of two stages. In stage I, there are no static connections (except for T4). This means that each subject, whether a proposer (blue dot) or a responder (pink dot), interacts with four randomly selected subjects in the opposite role. There are a total of 48 subjects, comprising 24 proposers and 24 responders. After stage I, responders in each group are ranked according to their *q* values, and the top six subjects are designated as leaders (represented by red stars in the diagram). The same 48 subjects participate in stage II, which takes place on a bipartite network consisting of two groups of nodes, with no intra-group connections. Proposers and responders are assigned to one of the two groups of nodes, respectively. We implement three treatments (T1, T2, and T3), each with distinct conditions. However, in stage II, the positions within the network remain fixed for all subjects throughout the stage. (T1) “Random” treatment: On a regular network where each node has four neighbors, proposers and responders are randomly assigned to the nodes. The leaders identified in stage I are placed on the nodes marked by stars. (T2) “Peripheral” treatment: On a heterogeneous network, proposers have four neighbors, as in T1, while responders are placed on six central nodes and 18 peripheral nodes. The central nodes each have seven neighbors, while the peripheral nodes have three neighbors. The leaders identified in stage I are positioned at six spatially adjacent peripheral nodes (marked by stars). (T3) “Central” treatment: The network structure is the same as in T2, but the leaders identified in stage I are placed on the six central nodes (marked by stars). Both the regular and the heterogeneous networks consist of the same number of links. (T4) Additional treatment: There are static network structures in both stages. The network structure in stage I is the same as in stage II of T1 (i.e., the random condition), while the network structure in stage II is the same as in stage II of T3 (i.e., the central condition).

A laboratory behavioral experiment is a controlled study conducted in a lab setting to investigate human decision-making and behavior under specified conditions. As mentioned above, our experiment consists of two stages. In the first stage (stage I), each subject participates in UGs with four randomly selected partners per round, with partners reshuffled after each round, ensuring no stable social ties. Half of the subjects are assigned the role of proposers and the other half the role of responders. At each round, proposers state their proposed offers, hereafter *p*, and responders state their minimum acceptable offers, hereafter *q*. The purpose of stage I is to identify leader responders, i.e., individuals who consistently demonstrate strong fairness preferences regardless of network position. In the second stage (stage II), the same subjects engage in UGs on a static bipartite network, where their partners remain fixed throughout. The network can be either homogeneous or heterogeneous. The goal of stage II is to determine whether fairness-preference leaders maintain their fairness behavior under different contexts and whether collective fairness can be influenced by network structures and individual fairness preferences. Our experiment includes three different treatments: (1) random, (2) peripheral, and (3) central, denoted as T1, T2, and T3, respectively. These treatments differ in network structures and the locations of responders in stage II (see Figure 1). In T1, fairness-preference leaders identified in stage I are randomly assigned to the responder nodes of a regular bipartite network in stage II. In T2, we conduct an unbiased comparison with T1 by constructing an inhomogeneous network with the same number of nodes and links as in T1. Specifically, all proposers in the network have four neighbors, but there are two types of responder nodes: central nodes with seven neighbors and peripheral nodes with three neighbors. Fairness-preference leaders identified in stage I are placed on peripheral nodes, which are spatially clustered in a local region in stage II. In T3, we use the same inhomogeneous network as in T2, but the leaders identified in stage I are positioned on central, influential nodes with a greater number of neighbors in stage II (see the methods section for more details on the experimental settings).

We also conduct an additional treatment, T4, to examine the robustness of the fairness-preference leader effect and the combined impact of network heterogeneity and individual fairness preferences on both individual and collective fairness (see Figure 1). Unlike T1 to T3, in T4, subjects participate in UGs on static networks in two stages. In stage I, we identify fair-preference leaders on a regular network with the same structure as that used in stage II of T1 (random condition). In stage II, the leaders identified in stage I occupy central nodes in a heterogeneous network with the same structure as that used in stage II of T3 (central condition).

### Allocation fairness and efficiency

**Figure 2:**
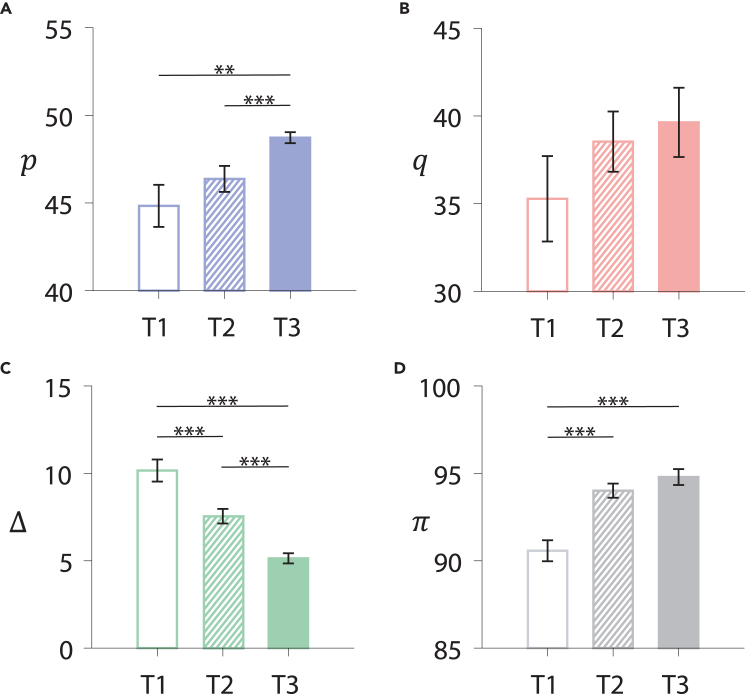
Allocation fairness and efficiency on networks (A) Average offer *p* of proposers, (B) average acceptance level *q* of responders, (C) average payoff difference Δπ between proposers and responders, and (D) allocation efficiency (see text for the definition) *π* in stage II of T1, T2, and T3. Averages are taken over the last 10 rounds of each group and error bars represent the standard error of the mean (SEM). The Wilcoxon signed-rank test was performed, with significance levels defined as follows: ∗p≤0.05, ∗∗p≤0.01, ∗∗∗p≤0.001.

We measure the collective fairness of subjects by examining the proposers’ offers (*p*) and the responders’ acceptance levels (*q*). In general, higher values of *p* and *q* reflect a greater level of fairness within the population. Figures 2A and 2B show that in stage II, the highest average values of *p* and *q* are achieved simultaneously in T3 (the central condition), while regular networks yield the lowest values of *p* and *q* in T1 (see also Table S1). These results suggest that an inhomogeneous structure promotes fairness more effectively than a regular network. Population fairness is influenced not only by network structure but also by the stability of fairness-preference behavior and the positions of the fairness-preference leaders. When fairness-preference leaders occupy influential nodes in T3, the potential of network heterogeneity is fully unlocked, yielding the highest fairness. As an additional measure of fairness, we have also examined the payoff difference, Δπ, between proposers and responders (see Note S1 for details). We observe the smallest Δπ in T3 (see Figure 2C), which is consistent with the results for *p* and *q*, providing evidence that coordinating individual diversity with social structure heterogeneity can promote fairness within populations.

Note that the mean value of p=47.9 in T3 is closely aligned with the perfectly fair resource distribution (p=50) and is significantly higher than previous experimental results that did not account for the combined effect of individual fairness preferences and network heterogeneity.^1^^,^^9^^,^^10^^,^^33^^,^^34^^,^^35^^,^^36^^,^^38^ Our findings indicate a motivational dynamic in social networks that supports the high level of human fairness necessary for large-scale cooperation. We also observe that the variance of *q* is significantly larger than that of *p* in both homogeneous and heterogeneous networks, suggesting that responder behavior is inherently different from proposer behavior, regardless of network structure.^38^^,^^46^

In addition to fairness, we also study allocation efficiency, which is also a concern in sociology and economics, as well as in many key fields, such as artificial intelligence, health care, and transportation.^47^^,^^48^^,^^49^^,^^50^ However, there is often a tension between allocation fairness and efficiency, where a stronger preference for fairness can lead to more rejections of unfair behavior, resulting in lower bilateral payoffs.^51^^,^^52^ We measure allocation efficiency by the average total payoff, *π*, received by a proposer and a responder. The larger the value of *π*, the higher the probability of successful assignments, and the greater the allocation efficiency. Figure 2D shows that the highest levels of allocation efficiency and fairness are achieved simultaneously in T3, with any tension between the two being resolved. Interestingly, in an evolutionary context, high allocation efficiency enhances group survival fitness and promotes fairness within populations. More detailed results on the evolution of allocation fairness and efficiency can be found in Figures S1 and S2.

### Individual strategies

**Figure 3:**
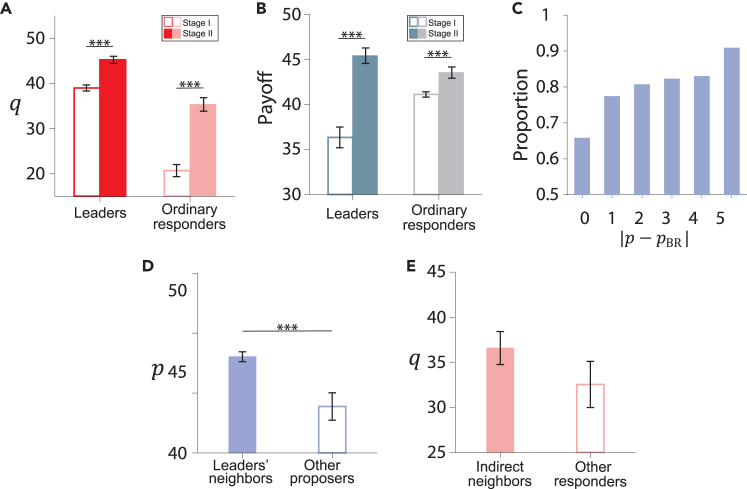
Individual behaviors and strategies (A) Average acceptance level *q* of leaders and ordinary responders in the two stages. (B) Average payoffs of leaders and ordinary responders in the two stages. (C) Fraction of proposers adopting the BR strategy in stage II, defined as the proportion of offers that fall within the deviation of $\left|p-p_{\text{BR}}\right|$. Here, $p_{\text{BR}}$ corresponds to the rigorous BR strategy and $\left|p-p_{\text{BR}}\right|$ denotes the deviation from this strategy, where 0≤p≤100. (D) Average offer *p* of the proposers who connect with leaders (leader’s neighbors) and that of the other proposers in stage II. (E) Average acceptance level *q* of the responders who share proposers with leaders (indirect neighbors) and that of the other ordinary responders. Error bars denote mean ± SEM. Averages are taken the last 10 rounds in stages I (A, B) and II. The Wilcoxon signed-rank test was performed, with significance levels defined as follows: ∗p≤0.05, ∗∗p≤0.01, ∗∗∗p≤0.001.

Having presented the results at the population level, we now turn to a discussion of individual strategies to uncover the underlying mechanisms that drive allocation fairness and efficiency. Figure 3A shows the fairness preferences of leaders in the two stages. The fairness-preference leaders identified in stage I maintain their high fairness behavior in stage II. This behavioral consistency suggests that their fairness preference is an inherent characteristic that governs their actions even under different contexts.^15^^,^^16^ In particular, Figure 3B shows that in stage I, where there are no ongoing social ties, leaders exhibit a strong fairness preference even when their payoffs are lower. Conversely, in stage II, the leader’s strong fairness preference becomes advantageous, resulting in payoffs higher than those of the other ordinary responders (see Figure 3B). The difference in leader payoffs between the two stages arises because the static connections in stage II allow leaders to punish unfair proposers. In contrast, in stage I, without static connections, unfair proposers are less frequently punished, as they change partners every round and only occasionally encounter leaders. More detailed results about the fairness-preference leaders are shown in Figures S2, S3, and S4.

Most proposers employ myopic best-response (BR) strategies to maximize their profits, considering the strategies of their neighbors from the previous round^53^^,^^54^ (see Note S2 for details). If we relax the strict definition of BR within a small range, more than 90% of proposers adopt self-interested BR strategies in stage II (Figure 3C). The BR behavior is common in stage II, regardless of whether the network structure is homogeneous or heterogeneous. Due to the punishment from the fairness-preference leaders, the BR strategy of their neighbors is to offer a high *p* value (Figure 3D). In contrast, in stage I, unfair proposers cannot be continuously punished due to the absence of a static structure. Therefore, the combination of the BR strategy and static network structure allows fairness-preference leaders to influence their neighbors and foster a high level of local fairness.

Unlike fair-preference leaders, ordinary responders exhibit conditional fairness behavior influenced by their local circumstances. For example, the indirect influence of leaders causes their *q* values to be significantly higher in stage II than in stage I (see Figure 3A). In stage I, the prevalence of low offers and the inability to punish prevent ordinary responders from insisting on fairness. Consequently, the changes observed in stage II occur because the proposers who are neighbors of leaders are compelled to increase their offers. Ordinary responders sharing proposers with leaders can then raise their acceptance levels, *q*, without reducing their payoffs (see Figure 3E). The results in both stages indicate that the behavior of ordinary responders is mixed: they aim to secure larger profits while also seeking fair treatment, but the profit motive takes precedence. The static network setup allows us to visualize this conditional fairness behavior. Further details on the evolution of individual behaviors and interaction patterns are presented in Figure S5.

### Mechanism underlying allocation fairness and efficiency

**Figure 4:**
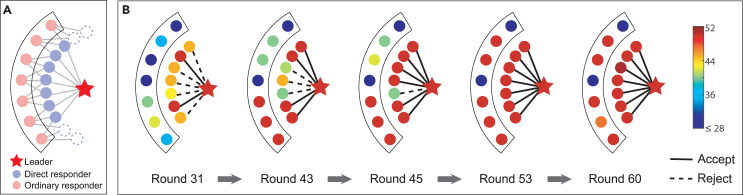
An illustration of the evolutionary process of allocation fairness and efficiency in stage II (A) The local structure of the leader, including direct proposers and indirect ordinary responders. The leader is represented by a star, with its direct neighbors being the proposers. The remaining individuals within the box are ordinary responders who share the same neighbors as the leader. (B) Five snapshots are presented, illustrating a typical evolutionary process of allocation fairness and efficiency in stage II. Links between direct proposers and indirect ordinary responders are omitted for clarity. Stage II begins in round 31. The color bar represents the value of *p* or *q*. The solid and dashed lines denote successful and unsuccessful resource allocations (i.e., offer accepted and splitting implemented), respectively. The leader not only increases the offers made by neighboring proposers but also influences the acceptance levels of indirectly neighboring responders.

Our main experimental observations are the fairness-preference leader effect, the widespread use of the BR strategy among proposers, and the conditional fairness behavior of ordinary responders, who prioritize payoff. Together, these three factors form a mechanism that ensures fairness in a heterogeneous network. Figure 4 illustrates a representative leader-driven evolutionary process of allocation fairness and efficiency. The leader maintains a strong commitment to fairness at the beginning of stage II (round 31) and plays a dominant role in the evolution. After several rounds, neighboring proposers come to realize that the leader is likely to maintain a high rejection value, *q*, regardless of the payoffs. From round 31 to round 45, the pressure for high fairness compels proposers using the BR strategy to gradually increase their offers, *p*, in order to avoid punishment from the leader (see Figure 4). With this increase in offers, from round 43 to round 53, most ordinary responders connected to proposers exhibit conditional fairness and gradually raise their *q* values. Finally, by round 60, a consensus on high group fairness is reached between proposers and responders in the local environment. As a result of this agreement, the success rate of resource allocation is high, leading to increased allocation efficiency. This evolutionary process is common in stage II, illustrating a general leader-driven interaction pattern and the indirect effects of networks on interactions. Leveraging stable social ties, leaders directly influence proposers and, through them, indirectly influence ordinary responders. Thus, in a heterogeneous network, placing leaders on central, influential nodes amplifies the leader effect, drives allocation fairness, and fully harnesses the ability of heterogeneous networks to enhance both allocation fairness and efficiency.

It is worth noting that ordinary responders behave like free riders in the sense that they rely on leaders to bear the cost of punishing unfair proposers. However, leaders, when supported by a static network, are effective in establishing a fair environment that encourages ordinary responders with conditional fairness behaviors to follow their lead and sustain fairness. As a result, free-riding behaviors that undermine fairness are mitigated by the combination of leaders and stable social ties. Since the majority of ordinary responders ultimately assist leaders in exerting higher pressure and greater threat of sanctions on unfair proposers, fairness norms are reinforced.

### Additional experimental evidence

**Figure 5:**
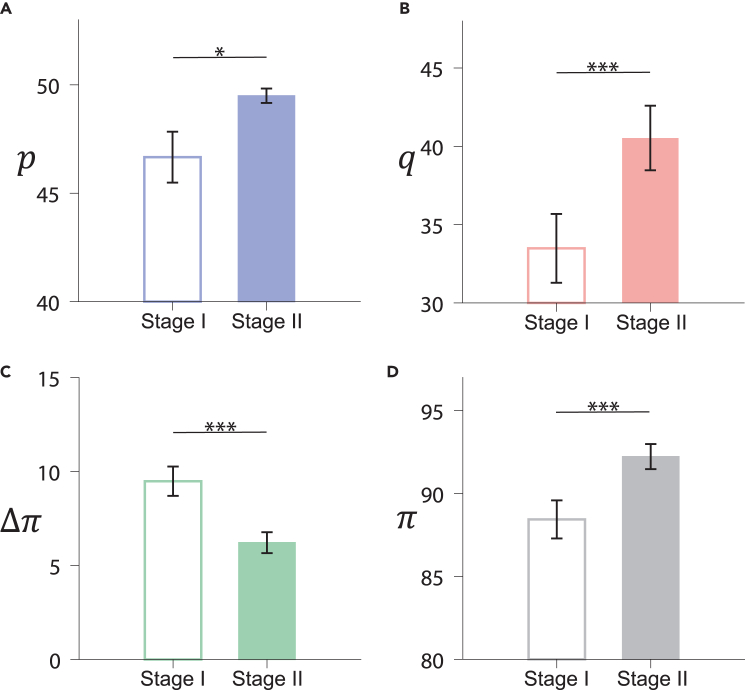
Allocation fairness and efficiency in T4 Experimental results of mean values and the standard error of the mean (SEM) of (A) offer *p* of proposers, (B) acceptance level *q* of responders, (C) payoff difference Δπ, and (D) allocation efficiency *π* in stage I and stage II in T4. The Wilcoxon signed-rank test was performed, with significance levels defined as follows: ∗p≤0.05, ∗∗p≤0.01, ∗∗∗p≤0.001.

To determine whether the leader effect depends on how leaders are identified in stage I, and whether this effect is general across networks, we conducted an additional treatment, T4, in which subjects play UGs on static networks over two stages, as shown in Figure 1. In stage I, we identify leaders on a regular network with the same structure as in stage II under the T1 (random condition). In stage II, leaders occupy central nodes in a heterogeneous network, similar to the structure in stage II under the T3 (central condition). We find that all fairness-preference leaders have high *q* values in both stages of T4, which is consistent with the results of T1, T2, and T3, providing additional experimental evidence for the general effect of leaders. The findings of T4 also provide direct evidence of the positive effect of network heterogeneity on fairness by comparing stage I and stage II with the same subjects. Interestingly, and similar to T1, T2, and T3, the heterogeneous network exhibits higher fairness than the homogeneous network, as shown in Figures 5A–5D that *p*, *q*, and *π* in stage II are larger than that in stage I, and Δπ in stage II is less than that in stage I (see Table 1 and Figure S6 for more details).

**Table 1:** Mean values/standard deviation of *p*, *q,*Δπ, and *π* in stage I and stage II of T4 experiments

	*p*		*q*	
	All 30 rounds	Last 10 rounds	All 30 rounds	Last 10 rounds

Stage I	46.66/8.13	46.86/8.30	33.48/15.22	35.86/15.28
Stage II	49.50/2.31	49.49/3.66	40.53/14.27	41.46/15.14

	Δπ		*π*	

	All 30 rounds	Last 10 rounds	All 30 rounds	Last 10 rounds
Stage I	9.48/5.40	8.02/7.21	88.44/7.96	91.61/8.94
Stage II	6.22/3.85	5.01/5.27	92.22/5.23	94.76/6.26

The results are obtained from different numbers of rounds.

### Evolutionary game model

We apply evolutionary game theory, incorporating individual preferences, to model UGs on networks with heterogeneous subjects. In general, we classify behaviors of proposers to two categories: fair (F) with fair sharing or rational (R) with self-interest in payoffs. To simplify our analyses, we assume polarization of the two categories, i.e., rational proposers offer a low proportion of resources *l* to responders, and fair proposers offer a high proposal *h* to responders.^32^ Akin to proposers, we also classify behaviors of responders to fair (F, reject low proposals) and rational (R, accept any proposals). In combination with the influence of fair preference, we define the utility of subjects with respect to both payoffs and (disadvantage) inequality aversion and construct a utility matrix. Specifically, we assume that the utility of responders resulting from inequality aversion is proportional to the payoff difference, and we introduce an inherent parameter *α* that characterizes the diversity of subjects in responding to inequality. Following the experiments, two types of responders are considered, leader responders who have a strong fairness preference $\alpha_{1}$ and ordinary responders who have a lower $\alpha_{2}$. Thus, the utility matrices of proposers vs. two types of responders can be defined, where the utility matrix of proposers vs. leader responders is

(Equation 1) $$\begin{array}{c} F\\ R \end{array}\left(\begin{array}{cc} F & R\\ 1-h,h & 1-h,h\\ 0,0 & 1-l,l-\alpha_{1}\left(1-2l\right) \end{array}\right)$$

and that of proposers vs. ordinary responders is

(Equation 2) $$\begin{array}{c} F\\ R \end{array}\left(\begin{array}{cc} F & R\\ 1-h,h & 1-h,h\\ 0,0 & 1-l,l-\alpha_{2}\left(1-2l\right) \end{array}\right)$$

The only difference between the two matrices is the parameter *α* that captures the perception of inequality of responders.

**Figure 6:**
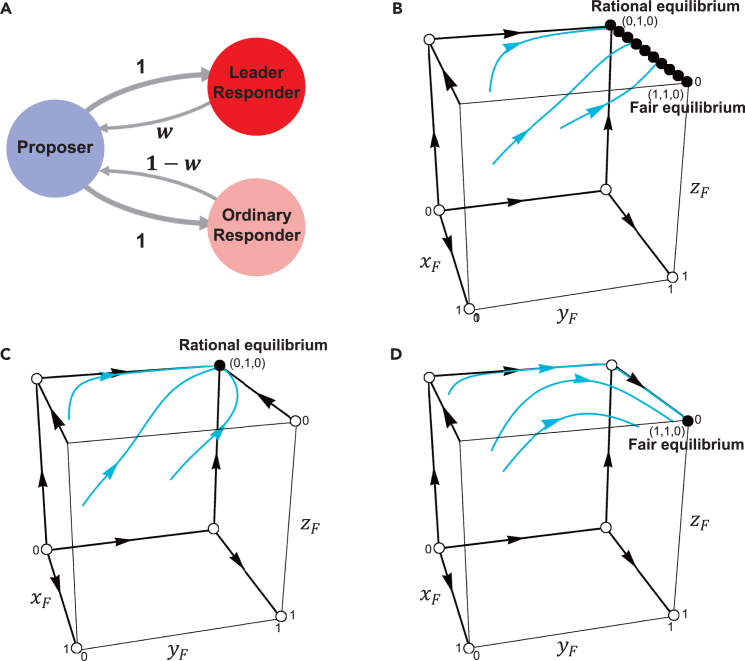
Evolutionary game model and phase portrait of the replicator dynamics (A) A mean-field approximation is applied to simplify the networked ultimatum game. The reduced system consists of three nodes: a proposer, a leader responder, and an ordinary responder. The values indicate the interaction weights between the nodes. (B) In the random condition, there exists a continuum of equilibria, with both the fair equilibrium $\left(x_{F},y_{F},z_{F}\right)=\left(1,1,0\right)$ and the rational equilibrium $\left(x_{F},y_{F},z_{F}\right)=\left(0,1,0\right)$ being neutrally stable. (C) In the peripheral condition, the rational equilibrium $\left(x_{F},y_{F},z_{F}\right)=\left(0,1,0\right)$ is globally stable. (D) In the central condition, the fair equilibrium $\left(x_{F},y_{F},z_{F}\right)=\left(1,1,0\right)$ is globally stable.

We then employ replicator dynamics to model the collective evolution of subjects affected by their interactions.^55^^,^^56^ To enable analytical results, we reduce the network system based on the mean-field approximation. As shown in Figure 6A, the simplified system consists of three nodes, i.e., a proposer, a leader responder, and an ordinary responder, representing three typical subjects in the original experimental network. The link in the original network is converted to the interaction weights in the reduced network. In particular, because the payoff of each subject from playing with his/her neighbors is normalized by his/her number of neighbors, in the reduced network the sum of either incoming or outgoing link weights should be one. The interaction weight from the lead responder to the proposer is denoted by *w*. We show in Note S3 that $w=\frac{1}{4}$ in the random condition, $w=\frac{3}{16}$ in the peripheral condition, and $w=\frac{7}{16}$ in the central condition. Let $x_{F}$, $y_{F}$, and $z_{F}$ be frequencies of *F* strategy in proposer population, leader responder population, and ordinary responder population, respectively. The replicator equations of the three nodes in the reduced network can be formulated as

$$\frac{dx_{F}}{dt}=x_{F}\left(1-x_{F}\right)\left(\left(1-l\right)\left(wy_{F}+\left(1-w\right)z_{F}\right)+l-h\right)$$

$$\frac{dy_{F}}{dt}=y_{F}\left(1-y_{F}\right)\left(1-x_{F}\right)\left(\alpha_{1}\left(1-2l\right)-l\right)$$

(Equation 3) $$\frac{dz_{F}}{dt}=z_{F}\left(1-z_{F}\right)\left(1-x_{F}\right)\left(\alpha_{2}\left(1-2l\right)-l\right)$$

The derivation and stability analysis of the replicator dynamics is shown in Note S3.

We now compare the evolutionary game model with the experimental data. As shown in Figures 6B and 6C, the equilibrium locations differ across the three conditions. In the random condition, there exists a continuum of neutrally stable equilibria that lies on the boundary $y_{F}=1$, which includes the fair and rational equilibria at the vertices, as well as other equilibria along the edge between the fair and rational equilibria, and the trajectories of Equation 3 will converge to this boundary. In the other two conditions, there is only one globally stable equilibrium: the rational equilibrium in the peripheral condition and the fair equilibrium in the central condition. Given the multiple equilibrium nature in the random condition, its behavioral evolution will be influenced by the initial state, potentially stabilizing at any of these equilibria. Furthermore, in the experiments, fairness-preference leader responders reject lower offers and ordinary responders accept lower offers, i.e., $y_{F}=1$ and $z_{F}=0$. Thus, we are particularly interested in the two equilibria $\left(x_{F},y_{F},z_{F}\right)=\left(1,1,0\right)$ and (0,1,0), where in the first, proposers provide fair proposal and in the second, provide rational proposal. In the experiments, responders are divided into two classes according to their average acceptance levels in stage I, where the acceptance levels for leader responders and ordinary responders are 40 and 20, respectively. Thus, *h* and *l* could be taken as 40% and 20%, respectively. Following the theoretical analysis in Note S3, inequality perceptions for leader and ordinary responders should satisfy $\alpha_{2}<\frac{1}{2}<\alpha_{1}$, and the fair equilibrium (1,1,0) (or the rational equilibrium (0,1,0)) is globally stable for $w>\frac{1}{4}$ (or $w<\frac{1}{4}$). Notice that $w=\frac{1}{4}$ in the random condition, there is a continuum of equilibria, where both the rational equilibrium $\left(x_{F},y_{F},z_{F}\right)=\left(0,1,0\right)$ and the fair equilibrium $\left(x_{F},y_{F},z_{F}\right)=\left(1,1,0\right)$ are neutrally stable (see Figure 6B). In the peripheral condition, $w=\frac{3}{16}<\frac{1}{4}$ and the rational equilibrium $\left(x_{F},y_{F},z_{F}\right)=\left(0,1,0\right)$ is globally stable (see Figure 6C). Finally, in the central condition, $w=\frac{7}{16}>\frac{1}{4}$ and the fair equilibrium $\left(x_{F},y_{F},z_{F}\right)=\left(1,1,0\right)$ is globally stable (see Figure 6D).

Taken together, our evolutionary model predicts that the fair equilibrium is globally stable in the central condition (T3), and the rational equilibrium is globally stable in the peripheral condition and the random condition. These equilibria indicate that the fairness level in the central condition is significantly higher than that in the other two conditions, which is in agreement with the experimental findings, as shown in Figure 2.

### Agent-based simulations

We further carry out agent-based simulations to explore how different degrees of network heterogeneity affect fairness within populations under the central and peripheral conditions. All bipartite networks for implementing agent-based simulations are composed of 240 nodes, where one half of nodes are occupied by proposers and the other half are occupied by responders. Proposers’ connections are homogeneous and each proposer has four responder neighbors. In contrast, responders’ connections are inhomogeneous, and a responder can have either three (i.e., peripheral nodes) or *k* (i.e., central nodes) proposer neighbors. In each simulation, we let k= 5, 6, 7, 8, or 9, respectively, in order to keep the total number of links unchanged in every simulation network. As a result, the numbers of responders with k=5, 6, 7, 8, or 9 neighbors are 60, 40, 30, 24, or 20, respectively. We use the coefficient of variation (CV) to quantify degree heterogeneity in a network, which is defined as the ratio of the standard deviation of the degree distribution to the network’s average degree. Thus, the *CV* of networks on the responder side with k=5,6,7,8, and 9 is $1/4,\sqrt{2}/4,\sqrt{3}/4,2\sqrt{2}/4$, and $\sqrt{5}/4$, respectively. Furthermore, we use the result of a regular network with identical k=4 as a baseline (random condition associated with a regular network).

**Figure 7:**
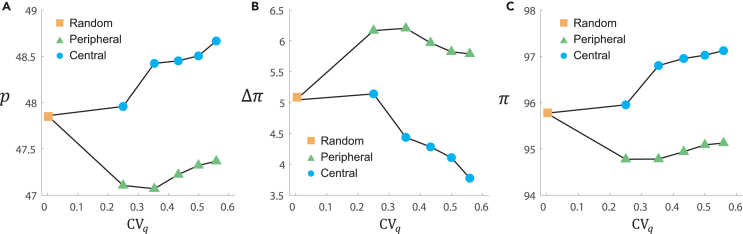
Agent-based simulation results on a variety of bipartite networks Each data point is plotted averaging over 1,000 simulations. In each simulation, we calculate the best-response offers of proposers based on the responder behavior sequences in the database. (A) *p*, (B) Δπ, and (C) *π* are obtained by averaging over the last 10 rounds. In analogy with experimental results, placing leaders on the central nodes (i.e., T3) can promote both allocation fairness and efficiency in heterogeneous networks.

To implement simulation, we assume that proposers use the best-response strategy according to our experimental findings. For responders, because of their heterogeneous and diverse behaviors, we build a database using the data of 144 responders in T1, T2, and T3, and choose behaviors of responders from the database. To be concrete, the database contains five copies of all responder behavior sequences obtained in our experiments. We randomly pick 120 sequences used in each simulation, representing 120 responders. By ranking the acceptance level *q* of these responders in stage I, we choose *x* responders of them as leaders who have the highest acceptance level *q* in stage I. Consistent with experimental settings, in the random condition, leaders are randomly distributed on the 120 (responder) nodes in a homogeneous network with k=4; in the peripheral condition, leaders are placed on peripheral nodes of a heterogeneous network only; in the central condition, leaders occupy central nodes of a heterogeneous network. Based on the responder behavior sequences in stage II, we calculate the best-response offers of proposers, and the three indices *p*, Δπ, and *π*. Simulation results are shown in Figures 7A–7C. In analogy with experimental results, placing leaders on the central nodes (i.e., the central condition) always promotes both allocation fairness and efficiency in heterogeneous networks with different degrees of heterogeneity, compared with random and peripheral conditions.

To further validate the robustness of our experimental findings, we perform one-shot UG simulations on a series of strongly heterogeneous networks, including BA-like bipartite scale-free networks and nine empirical bipartite networks. The details of how these BA-like bipartite scale-free networks are generated can be found in Note S4. We refer to the distribution of acceptance levels presented in Mei et al. (2024) to generate acceptance levels of responders.^57^ This distribution, derived from behavioral economic game data involving 88,595 subjects across 15,236 sessions, is highly versatile and applicable in diverse contexts, facilitating a more comprehensive analysis of the combined effects of network heterogeneity and fairness preferences. For simplicity and without loss of generality, we randomly generate the acceptance levels based on this distribution, which only includes the values 0, 10, 20, 30, 40, and 50 with probabilities of 0.18, 0.06, 0.11, 0.17, 0.24, and 0.24, respectively. We first rank the nodes in descending order by degree and then assign the generated acceptance levels in random, ascending, or descending order to the nodes in the network, corresponding to random, peripheral, and central conditions, respectively. One-shot UG simulations are then conducted to generate proposers’ offers using the best-response strategy, assuming that their responder neighbors do not alter their acceptance levels. Table 2 presents the simulation results for bipartite scale-free networks with varying sizes, average degrees, and degree heterogeneity. Overall, network heterogeneity can amplify the influence of fairness-preference neighbors, leading to higher values of *p* and *π*, as well as a smaller value of δπ. Additionally, we conduct further simulations on nine empirical bipartite networks, with the results provided in Table S2. Across both BA-like bipartite scale-free networks and empirical bipartite networks, the simulation results show that collective fairness and allocation efficiency are highest under the central condition, followed by the random condition, while the peripheral condition yields the lowest performance.

**Table 2:** Simulation results of *p*, *π*, and Δπ across various bipartite scale-free networks with different network sizes *N*, average degrees of responders ${\left\langle k\right\rangle }_{q}$, and coefficient of variation ${\text{CV}}_{q}$ of responders

*N*	${\left\langle k\right\rangle }_{q}$	${\text{CV}}_{q}$	Random/peripheral/central		
			*p*	*π*	Δπ
1,000	3.85	1.06	40.92/34.02/43.45	96.43/89.25/99.64	14.30/36.77/5.80
	7.61	0.87	43.68/36.03/46.25	96.50/88.12/98.71	8.79/27.92/3.74
	14.88	0.71	46.18/38.68/48.13	96.68/87.16/99.17	5.88/24.94/1.59
	28.62	0.58	47.84/41.42/49.18	99.12/88.74/99.36	1.21/20.85/1.07
10,000	3.97	1.43	40.46/33.63/43.80	97.38/89.20/99.39	13.44/36.69/6.86
	7.91	1.21	43.85/45.61/46.40	96.82/86.92/98.97	7.81/30.04/3.41
	15.75	1.05	46.14/37.62/48.22	97.40/86.66/99.12	4.50/26.91/1.56
	31.25	0.93	47.93/39.99/49.37	97.39/87.65/99.70	3.72/22.51/0.47
	61.68	0.82	48.94/41.61/49.72	98.87/87.63/99.85	1.44/20.98/0.19
100,000	3.99	1.77	40.60/33.65/43.38	97.42/89.22/99.35	13.00/36.94/6.92
	7.98	1.54	43.84/35.53/46.41	96.82/86.87/98.92	7.87/32.29/3.31
	15.95	1.36	46.16/37.48/48.25	97.20/85.79/99.14	4.83/28.51/1.61
	31.85	1.24	47.93/39.53/49.39	98.12/85.95/99.65	2.70/25.65/0.55
	63.53	1.13	48.90/41.12/49.74	98.93/86.56/99.83	1.34/23.40/0.21
	126.49	1.04	49.58/42.30/49.93	99.60/87.26/99.95	0.47/21.72/0.06

The bipartite networks consist of an equal number of nodes on each side. Each data point represents the average of 100 network simulations.

The computational results from the agent-based simulations on artificial networks with weak and strong heterogeneity as well as empirical networks provide further support for the hypothesis that social diversity—the combination of individual differences in fairness preferences and networks with more influential locations—promotes allocation fairness and efficiency in heterogeneous networks.

## Discussion

In this study, we have provided evidence from two-stage UG experiments demonstrating that the combined effect of network heterogeneity and individual fairness preferences can promote fairness within populations. Furthermore, we have shown that this effect is significantly amplified when leader responders with a strong fairness preference occupy influential nodes. This is because static social ties enable leaders to punish neighboring proposers who exhibit unfair behavior by rejecting their offers. As a result, proposers are compelled to behave fairly, which in turn triggers conditional fairness behavior in ordinary responders. When fairness-preference leaders occupy influential nodes with more connections, their ability to drive fairness is amplified, ensuring high fairness between proposers and responders. In this way, high allocation efficiency is achieved. Our mean-field replicator dynamics model and agent-based simulations further verify that the dominance of fairness driven by leaders is prevalent in networks with varying degrees of heterogeneity.

Since social diversity is ubiquitous, it is crucial to examine whether the mechanisms and interaction patterns observed in our experiments can be applied to other networked games.^11^ Note, for example, that a leader’s rejection of unfair offers is analogous to costly punishment for defection in the prisoner’s dilemma game.^58^^,^^59^ Thus, in the prisoner’s dilemma game or any public goods game involving punishment, leaders use punishment to promote cooperation.^60^^,^^61^^,^^62^^,^^63^ When leaders occupy highly connected nodes, their impact on cooperation is amplified, leading to a higher level of cooperation in heterogeneous networks. Similarly, social diversity plays a role in trust games,^64^ coordination games,^53^ and in the dynamics of many other games.^55^^,^^56^ This phenomenon also occurs in experiments where robots are used to coordinate social networks, highlighting the importance of network structure and leadership in fostering coordination.^65^ The key elements of this mechanism, present in all these examples, are the same as those identified in our work: social diversity (individual preference differences combined with network heterogeneity) and a procedure for punishing less prosocial individuals.

Given that our main conclusions apply to static networks, an important additional discussion pertains to the ability of individuals to disengage from unfair partners and select new ones.^66^^,^^67^^,^^68^^,^^69^^,^^70^^,^^71^^,^^72^ Whether an individual maintains a partnership or forms a new one is strongly influenced by the associated costs.^69^ When the cost of switching partners is high and there is no information about partner reputations, individuals tend to maintain their current connections and avoid change, consistent with our experimental stage II setting. However, when the cost of switching partners is low, the network structure evolves, stable social ties emerge, and the level of interaction fairness increases. In this context, link-cutting can be viewed as an additional form of punishment: leaders not only reject unfair offers but also prevent unfair proposers from contacting them again. Over time, fairness-preference leaders who use this mechanism attract an increasing number of stable connections, ultimately forming the influential nodes that characterize our experimental central condition. Thus, in both static and adaptive social networks, our experimental results provide valuable insights into the emergence and evolution of fairness.

Interestingly, our experiments raise a number of further questions about human fairness. While the behavior of leaders, ordinary responders, and proposers in social networks is clearly observed in the experiment, the psychological and biological foundations of these behaviors remain unclear. What are the evolutionary roots of leaders’ decisions to reject low payoffs? Why do only proposers adopt the best-response strategy? We note that if responders used this strategy, they would accept any offer, yet this is practically never observed. Why are there no proposer leaders who, by insisting on their decisions, would push responders to accept unfair offers? What is the effect of asymmetrical roles on decision-making? If we allow leader responders to act as proposers in stage II, will the results differ from those when ordinary proposers take this role? These questions not only call for further experimental validation but also require deeper theoretical exploration. In particular, our replicator model only provides a qualitative interpretation for the experimental results. Therefore, frameworks such as stochastic game theory^34^^,^^73^ could provide valuable insights into the strategic evolution of decision-making in social networks, helping to explain the persistence of seemingly suboptimal behaviors and the mechanisms underlying leadership dynamics.

Also, our finding regarding the indirect influence of fairness-preference leaders on ordinary responders opens up exciting avenues for exploring how higher-order networks shape fairness and cooperation, while also raising new questions.^28^ For example, could leaders in high-order networks amplify their influence by leveraging the collective behavior of indirect social ties, thereby strengthening fairness norms within larger groups? How do these multi-layered interactions affect the persistence of fairness in scenarios where direct punishment or reward mechanisms may not be sufficient? These questions suggest that fairness and cooperation may be shaped differently when considering higher-order interactions, adding complexity to the dynamics we have observed. In this context, our experiments provide valuable insights into how social diversity and network structure influence fairness, cooperation, and decision-making, and point to the need for further exploration into the impact of higher-order networks.

Furthermore, we present evidence through a combination of experiments, theoretical analysis, and simulations. Our experiments are conducted on weakly heterogeneous networks, providing a controlled environment to explore the underlying dynamics. While our simulation results suggest that strongly heterogeneous networks may amplify the influence of fairness preferences, further experiments could reveal the extent and mechanisms underlying this effect. How might the structural differences between weakly and strongly heterogeneous networks influence the role of fairness preferences in shaping collective behavior? Could the dynamics of fairness preferences and collective fairness vary across different types of network structures and network sizes? Could fairness preferences manifest differently in student-dominated vs. more diverse participant pools, and what implications might this have for the generalizability of experimental findings? Addressing these questions could deepen our understanding of how network heterogeneity strength and individual preference affect collective fairness behavior.

Last but not least, our experiments, theoretical analysis, and simulations suggest that collective fairness behavior can be “shaped” by leveraging network structures and individual preferences. However, real-world networks may involve multiple layers of interaction, with feedback loops, evolving relationships, and broader societal constraints,^74^^,^^75^^,^^76^ all of which are typically not accounted for in controlled experiments. Therefore, while the insights from this study are valuable, the application of these results to broader societal contexts requires careful consideration of the differences in network dynamics, participant experiences, and external factors that influence real-world decision-making. Future studies should aim to bridge this gap by exploring the effects of these additional complexities in more naturalistic settings.

## Methods

### Experimental setting

The use of human subjects in our research was approved by the School of Systems Science, Beijing Normal University, and all participants provided informed consent prior to their involvement. Since students are often considered a “convenience sample” in laboratory behavioral experiments,^77^^,^^78^^,^^79^^,^^80^ a total of 384 undergraduate students were recruited to participate, with four experimental treatments: random (T1), peripheral (T2), central (T3), and additional (T4). Each treatment consisted of two groups, with 48 subjects per group. Within each group, half of the participants were assigned the role of proposers, and the other half were assigned as responders. Their roles remained fixed throughout the experiment once assigned. Both stage I and stage II consisted of 30 rounds. In each round, each subject made a single decision (either an offer or an acceptance level, depending on their role) for all of their partners. Specifically, proposers made the same offer *p* (0≤p≤100) to all their direct-neighbor responders, while responders maintained the same minimum acceptance level *q* (0≤q≤100) to all of their direct-neighbor proposers. In stage I, subjects were reshuffled on the network after each round, resulting in changing neighbors and the absence of static links. At the end of stage I, we ranked the 24 responders based on their *q* values and selected the six responders with the highest *q* values as leaders. In stage II, the subjects were placed on the nodes of a static bipartite network, where each proposer’s neighbors were responders and vice versa. Figure 1 illustrates the network configurations and the locations of the leaders under the different treatments.

At the beginning of each round, every subject receives information from the previous round, including their own behavior and score, as well as the behaviors of their partners (i.e., direct neighbors). A subject’s score in a round is the average of the points obtained across all their interactions. To simplify the decision-making process and eliminate any reputation effects, partner behaviors were ranked in descending order in each round and made available to each subject. For more details on the experimental design, refer to Notes S5 and S6, Figure S7, and Table S3.

## Resource availability

### Lead contact

Requests for further information and resources should be directed to and will be fulfilled by the lead contact, Boyu Zhang (zhangby@bnu.edu.cn).

### Materials availability

Materials are available from the lead contact upon reasonable request.

### Data and code availability

Our raw data and agent-based simulation code used in this study are publicly available at OSF (https://doi.org/10.17605/OSF.IO/Q3ETM).^81^

## Acknowledgments

X.H. acknowledges support from the 10.13039/501100001809National Natural Science Foundation of China under grant nos. 72288101 and 72371016. B.Z. acknowledges support from the Beijing Natural Science Foundation under grant no. Z220001, the 10.13039/501100012166National Key Research and Development Program of China under grant no. 2020YFA0712900, and the 10.13039/501100001809National Natural Science Foundation of China under grant nos. 72131003 and 71922004. A.S. acknowledges support from grant PID2022-141802NB-I00 (BASIC) funded by 975 MCIN/AEI/10.13039/501100011033 and by “ERDF A way of 976 making Europe” and from grant MapCDPerNets-Programa 977 Fundamentos de la Fundación BBVA 2022.

## Author contributions

X.H., B.Z., S.C., A.S., W.-X.W., and H.E.S. designed the research; X.H., B.Z., S.M., S.C., and W.-X.W. performed the research; X.H., B.Z., S.M., A.S., and W.-X.W. analyzed data, proposed the model, and conducted simulations; and X.H., B.Z., S.C., A.S., W.-X.W., and H.E.S. wrote the paper.

## Declaration of interests

The authors declare no competing financial interests.

## Declaration of generative AI and AI-assisted technologies in the writing process

The authors acknowledge the use of ChatGPT to assist in polishing the language of this paper. All content has been reviewed and edited under the authors’ oversight and control. No artificial intelligence tools were used for idea generation, data analysis, or development of research findings.

## Supplemental information

Document S1. Figures S1–S7, Tables S1–S3, and Notes S1–S6

Document S2. Article plus supplemental information
