# Supplementary material for: Coordination of network heterogeneity and individual preferences promotes collective fairness
Source: Patterns (N Y). 2025 Jun 16;6(11):101293. doi: 10.1016/j.patter.2025.101293 (PMC12664973; doi:10.1016/j.patter.2025.101293)
Supplement: Document S1. Figures S1–S7, Tables S1–S3, and Notes S1–S6 [file mmc1.pdf]

Patterns, Volume *6*

Supplemental information

Coordination of network heterogeneity and individual preferences promotes collective fairness

Xiao Han, Shangmei Ma, Wen-Xu Wang, Angel Sánchez, H. Eugene Stanley, Shinan Cao, and Boyu Zhang

Supplementary Information for

Coordination of network heterogeneity and individual preferences promotes collective fairness

Xiao Han[a], Shangmei Ma[b], Wen-Xu Wang[b], Angel Sánchez[c,d], H. Eugene Stanley[e], Shinan Cao*[f], and Boyu Zhang†[g]

[a]School of Systems Science, Beijing Jiaotong University, Beijing 100044, People's Republic of China

[b]School of Systems Science, Beijing Normal University, Beijing 100875, People's Republic of China

[c]Grupo Interdisciplinar de Sistemas Complejos (GISC), Departamento de Matemáticas, Universidad Carlos III de Madrid, Spain

[d]Instituto de Biocomputación y Física de Sistemas Complejos (BIFI), Universidad de Zaragoza, Zaragoza 50018, Spain

[e]Center for Polymer Studies and Department of Physics, Boston University, Boston, MA 02215

[f]School of Finance, University of International Business and Economics, Beijing 100029, People's Republic of China

[g]School of Mathematical Sciences, Beijing Normal University, Beijing 100875, People's Republic of China

*shinancao@uibe.edu.cn

†zhangby@bnu.edu.cn

Contents

1 Supplementary Note 1: Measuring fairness and efficiency 3

2 Supplementary Note 2: Evaluating self-interested behaviours 3

3 Supplementary Note 3: An evolutionary game model 4

4 Supplementary Note 4: Bipartite scale-free network 6

5 Supplementary Note 5: Experimental settings 6

6 Supplementary Note 6: Experimental instructions 7

7 Supplementary Figures 10

8 Supplementary Tables 17

9 Supplementary References 19

1 Supplementary Note 1: Measuring fairness and efficiency

We conducted three distinct treatments of networked UG experiments: the random condition (T1), the peripheral condition (T2), and the central condition (T3). Four indices, p, q, $\Delta\pi$ and π are used to examine the combined effects of network structures and individual fairness preferences on fairness and allocation efficiency within population. p and q are the offer of proposer and the acceptance level of responder, respectively. $\Delta\pi$ denotes the payoff difference between two neighboring proposer and responder. Suppose that there is a link between proposer i and responder j, where their payoffs are π_i and π_j, respectively. Then $\Delta\pi_{ij}$ is defined as $|\pi_i - \pi_j|$, and $\Delta\pi$ is the average of all $\Delta\pi_{ij}$. π denotes the average total payoff. Suppose that there is a link between proposer i and responder j. Then π_{ij} is defined as $\pi_i + \pi_j$, and π is the average of all π_{ij}.

p, q and $\Delta\pi$ measure fairness within populations, and π measures social efficiency (e.g., $\pi = 0$ if all offers are rejected and $\pi = 100$ if all offers are accepted). We examine the time evolution of these indices across the two stages of the experiment (see Figures S1-S2). In Stage I, the 3 treatments have the same setting. We then aggregate the results of T1, T2, and T3, and obtain that the mean values of p, q, $\Delta\pi$, and π are about 43.3, 26.2, 12.5, and 90.1, respectively. These findings are consistent with those observed in many other UG experiments in the literature, in which on average $p \approx 40$ and $q \approx 30$. In contrast, p, q, and $\Delta\pi$ in Stage II of T1, T2 and T3 are larger than those in Stage I (see Figure S1 and Table S1). These results suggest that static network structures can promote fairness compared to dynamic interactions. In particular, placing leaders on central nodes (i.e., T3) yields the highest levels of both fairness and efficiency.

2 Supplementary Note 2: Evaluating self-interested behaviours

We employed a rigorous test to identify whether participants were self-interested. Participants who adopted the optimal strategy in response to the strategies of their neighbors in the previous round were classified as self-interested. For self-interested responders, the optimal strategy is to accept all proposals from their neighbors. For self-interested proposers, the optimal strategy in each round is to offer the amount that maximizes their payoffs, based on the minimum acceptance thresholds of their neighbors in the previous round [1, 2]. For a proposer with k neighbors ($k = 4$ in our all experiments) whose minimum acceptance levels in the previous round were respectively $q_1, ..., q_k$ (with $q_1 < \cdots < q_k$), the best strategy was

$$p = \text{argmax}_{q_i}\{i \times (100 - q_i)/k\},$$

where $i \times (100 - q_i)/k$ was the payoff if the proposers offered q_i. We found that the proportion of rational proposers gradually increased and eventually exceeded half of the population in all groups. It is important to note that the definition of self-interested behavior was very strict.

If we were to slightly relax this definition, a larger fraction of proposers would be classified as self-interested, as show in Figure S5.

3 Supplementary Note 3: An evolutionary game model

We employ replicator dynamics to model the evolution of subjects influenced by their interactions. To enable analytical results, we simplify the network system using the mean-field approximation. As shown in Figure 6A, the reduced system consists of three nodes: a proposer, a leader responder, and an ordinary responder, representing three typical players in the network. The links in the original network are converted into interaction weights in the reduced network. Specifically, because each subject's payoff from interactions with neighbors is normalized by their number of neighbors, the sum of either incoming or outgoing link weights in the reduced network must equal one. Let the interaction weight from the leader responder to the proposer be denoted by w. The approximation principle for different network conditions is as follows:

(1) Random condition: In this condition, 6 out of 24 responders are designated as leaders, and each responder has 4 neighbors. Therefore, 24 out of 96 links connect proposers and leader responders, while the remaining 72 links connect proposers and ordinary responders. As a result, in the reduced network, the interaction weight from the leader responder to the proposer is one-third of the weight from the ordinary responder, i.e., $w = \frac{1}{4}$.

(2) Peripheral condition: Since 18 out of 96 links connect proposers and leader responders, while the remaining 78 links connect proposers and ordinary responders, in the reduced network the interaction weights from the leader responder and the ordinary responder to the proposer are $w = \frac{3}{16}$ and $1 - w = \frac{13}{16}$, respectively.

(3) Central condition: Since 42 out of 96 links connect proposers and leader responders, while the remaining 54 links connect proposers and ordinary responders, in the reduced network the interaction weights from the leader responder and the ordinary responder to the proposer are $w = \frac{7}{16}$ and $1 - w = \frac{9}{16}$, respectively.

Next, we formulate the replicator dynamics equations for the reduced network system. Let x_F, y_F, and z_F denote the frequencies of F strategy in proposer population, leader responder population, and ordinary responder population, respectively. Using the utility matrices, we can

then calculate the expected payoffs of subjects with different types and strategies:

$$\begin{aligned}
E_x(F) &= 1-h \\
E_x(R) &= w(1-l)(1-y_F)+(1-w)(1-l)(1-z_F) \\
E_y(F) &= x_F h \\
E_y(R) &= (1-x_F)(l-\alpha_1(1-2l))+x_F h \\
E_z(F) &= x_F h \\
E_z(R) &= (1-x_F)(l-\alpha_2(1-2l))+x_F h,
\end{aligned}$$

where $E_x(F)$ and $E_x(R)$ are the expected payoffs of proposers with F and R strategies, respectively, $E_y(F)$ and $E_y(R)$ are the expected payoffs of leader responders with F and R strategies, respectively, $E_z(F)$ and $E_z(R)$ are the expected payoffs of ordinary responders with F and R strategies, respectively, and w is the interaction weight from the lead responder to the proposer.

The replicator equations for the three nodes in the reduced network can be formulated as

$$\begin{aligned}
\frac{dx_F}{dt} &= x_F(1-x_F)((1-l)(wy_F+(1-w)z_F)+l-h) \\
\frac{dy_F}{dt} &= y_F(1-y_F)(1-x_F)(\alpha_1(1-2l)-l) \\
\frac{dz_F}{dt} &= z_F(1-z_F)(1-x_F)(\alpha_2(1-2l)-l)
\end{aligned}$$

Notice that

$$\begin{aligned}
\frac{dy_F}{dt} &> 0 \text{ for } \alpha_1(1-2l)-l>0, \\
\frac{dy_F}{dt} &< 0 \text{ for } \alpha_1(1-2l)-l<0, \\
\frac{dz_F}{dt} &> 0 \text{ for } \alpha_2(1-2l)-l>0, \\
\frac{dz_F}{dt} &< 0 \text{ for } \alpha_2(1-2l)-l<0,
\end{aligned}$$

the above dynamic system has no interior equilibrium in general. Moreover, it has eight boundary equilibria, where each of x_F, y_F, and z_F can be taken as either 0 or 1. In the experiments, leader responders reject lower offers and ordinary responders accept lower offers, i.e., $x_F = 1$ and $y_F = 0$. Thus, we are particularly interested in the stability conditions of two equilibria $(x_F, y_F, z_F) = (1, 1, 0)$ and $(0, 1, 0)$, where in the first proposers provide fair proposal and in the second provide rational proposal. Notice that the evolutionary directions of y_F and Z_F are independent of x_F, $(1, 1, 0)$ is locally stable (we refer it as fair equilibrium) if and only if

$$\alpha_2 < \frac{l}{1-2l} < \alpha_1 \text{ and } w > \frac{h-l}{1-l},$$

and $(0, 1, 0)$ is locally stable (we refer it as rational equilibrium) if and only if

$$\alpha_2 < \frac{l}{1-2l} < \alpha_1 \text{ and } w < \frac{h-l}{1-l}.$$

In addition, the inexistence of interior equilibrium implies that the locally stable boundary equilibria are also globally stable. The meanings of these conditions are clear. Condition $\alpha_2 < \frac{l}{1-2l} < \alpha_1$ means that leader responders should have a higher inequality aversion than ordinary responders and always reject unfair offers. Condition $w > \frac{h-l}{1-l}$ implies that a higher interaction weight from the leader responder to the proposer promote the stability of the fair equilibrium $(1, 1, 0)$.

4 Supplementary Note 4: Bipartite scale-free network

We adopt the edge-rewiring process in [3] to generate BA-like bipartite scale-free networks. First, we generate two separate Barabàsi-Albert subgraphs using the standard preferential attachment algorithm [4], where both subgraphs have the same network size and the number of edges. Denote two two subgraphs as Subgraph 1 and Subgraph 2, respectively. Then, we rewire all edges to connect nodes across the two subgraphs. In a rewiring, one edge connecting nodes i and j in Subgraph 1 and one edge connecting nodes k and l in Subgraph 2 are randomly chosen. We remove the two chosen edges and introduce two new edges, (i, k) and (j, l), linking nodes between the two subgraphs. The edge-rewiring process preserves the degree distribution of the network, as each node maintains its original number of connections. Furthermore, the structural properties of the individual subgraphs remain intact in terms of node degree, ensuring that the overall characteristics of the original BA subgraphs are retained.

5 Supplementary Note 5: Experimental settings

We implement 4 conditions of networked UG experiments in computer labs of Beijing Normal University, where each condition consists of 2 groups with 48 subjects in each group. Detailed settings are shown in Table S3. All of 384 subjects were freshmen and sophomores recruited from Beijing Normal University without taking classes of game theory and economy. Interactions among subjects are anonymous. Frosted glass dividers ensured that the students could not see each other. We built the experimental platform by using PHP, mySQL and javascript, and operate the platform programs on the server.

Before beginning the formal experiments, we spent 20 minutes introducing the experiment to all participants. This included explaining the rules and objectives of the game, as well as providing an overview of the feedback displayed on the screen interface. All participants received written instructions in Chinese (see Supplementary Note 6 for a translation). Each formal experiment consisted of 60 rounds, with 30 rounds in Stage I and 30 rounds in Stage II. Participants were not informed of the total number of rounds to avoid potential end-round effects. There was no time limit for decision-making, though participants were encouraged to submit their decisions within 30 seconds. The experiment lasted approximately 40 minutes.

Upon completion, each participant's total score was converted to Chinese Yuan at a rate of 50 : 1. This plus 20 Chinese Yuan is his/her final income. The average income in T1 was 73.1 Yuan (with a minimum of 57 and a maximum of 85), in T2 was 74.2 Yuan (with a minimum of 57 and a maximum of 89), in T3 was 74.8 Yuan (with a minimum of 62 and a maximum of 83), and in T4 was74.0 Yuan (with a minimum of 59 and a maximum of 80).

6 Supplementary Note 6: Experimental instructions

Instruction for T1

Welcome, and thank you for participating in this experiment. Please read the instructions carefully. If you have any questions, feel free to raise your hand, and one of the experimenters will come to assist you. From this point onward, communication with other participants is not allowed. Please turn off your mobile phone. The experiment consists of two stages: Stage I and Stage II. Your scores will depend on both your own decisions and the decisions made by the participants related to you.

$$\text{Your total income} = \text{show up fee (20 Yuan)} + \text{your total scores} \times 0.02 \text{ Yuan}.$$

1. *The basic game:*

There are two types of players: Player 1 and Player 2. Player 1 makes an offer on how to split 100 tokens, and Player 2 decides whether to accept or reject the offer. If Player 2 accepts, the tokens are divided according to Player 1's proposal. If Player 2 rejects, both players receive 0 tokens. In this experiment, you will play only one role: either as the proposer or as the responder.

2. *Rules of the game—stage I*

(1) At the beginning of the experiment, your role will be assigned randomly and will remain the same throughout the experiment.

(2) At the start of each round, you will randomly encounter 4 other participants, each playing a different role.

(3) In each round, Player 1 will play the basic game with each of their related players by inputting a value p, which represents the number of tokens given to each partner.

(4) In each round, Player 2 can decide whether to accept each of his/her related players' offer by inputting a value q, where an offer not less than q will be accepted.

(5) After all participants have submitted their values, the system will calculate your score. Your score = (your tokens)/(the number of partners).

2.1. *An example of Player 1*

(1) Player 1 plays with 4 Player 2s.

(2) Suppose that Player 1 gives p tokens to each player 2, and the acceptance levels of the 4

player 2s are $q_1 > q_2 > q_3 > q_4$.
(3) If $q_1 > q_2 > p \geq q_3 > q_4$, then two player 2s ($q_3$ and q_4) accept the offer. Player 1 gets $(200 - 2p)$ tokens and the score is $(200 - 2p)/4$ (4 is the number of partners).
2.2. *An example of Player 2*
(1) Player 2 play with 4 Player 1s.
(2) Suppose that the acceptance level of Player 2 is q, and the offers made by the 4 player 1s are $p_1 > p_2 > p_3 > p_4$.
(3) If $p_1 > p_2 > q \geq p_3 > p_4$, then two offers ($p_1$ and p_2) are accepted. Player 2 gets $(p_1 + p_2)$ tokens and the score is $(p_1 + p_2)/4$ (4 is the number of partners).
3. *Rules of the game—stage II*
(1) The game rules of stage II are the same as that in stage I.
(2) Your role in stage II is the same as that in stage I.
(3) In the beginning of stage II, your related players will be randomly reassigned.
(4) For Player 1, you play the game with 4 fixed Player 2. For Player 2, you play the game with 4 fixed Player 1.
(5) After all the participants submit their value, the system will calculate your score.

$$\text{Your score} = (\text{your tokens})/(\text{number of partners}).$$

4. *Payment*

$$\text{Your total income} = \text{show up fee (20 Yuan)} + \text{ your total score } \times 0.02 \text{ Yuan.}$$

Instruction for T2

3. *Rules of the game—stage II*
(1) The game rules of stage II are the same as stage I.
(2) Your role in stage II is the same as stage I.
(3 In the beginning of stage II, your related players will be randomly reassigned.
(4) For Player 1, you play the game with 4 fixed Player 2. For Player 2, you play the game with 3 or 7 fixed Player 1.
(5) After all the participants submit their value, the system will calculate your score.

$$\text{Your score} = (\text{your tokens})/(\text{number of partners}).$$

[The rest parts are same as T1.]

Instruction for T3

3. *Rules of the game—stage II*
(1) The game rules of stage II are the same as stage I.
(2) Your role in stage II is the same as stage I.

(3) In the beginning of stage II, your related players will randomly reassign.
(4) For Player 1, you play the game with 4 fixed Player 2. For Player 2, you play the game with 3 or 7 fixed Player 1.
(5) After all the participants submit their value, the system will calculate your score.

$$\text{Your score} = (\text{your tokens})/(\text{number of partners}).$$

[The rest parts are same as T1.]

Instruction for T4

3. *Rules of the game—stage I*
[This part is same as are same as *Rules of the game—stage II* of T1.]

[The rest parts are same as T3.]

7 Supplementary Figures

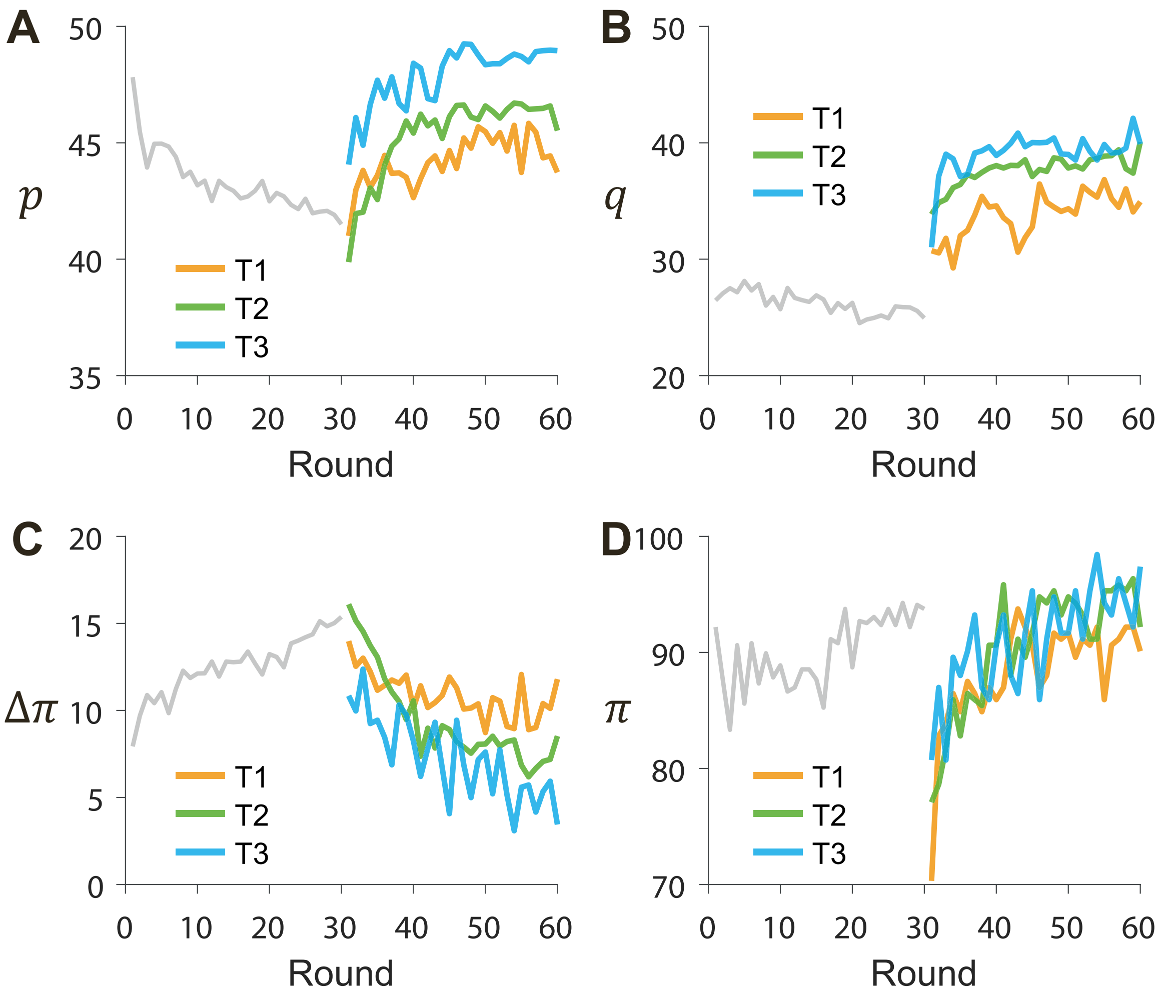

Supplementary Figure S1: Time evolution of allocation fairness and efficiency in T1, T2 and T3. **(A)** Time evolution of the mean values of p. In Stage I, p decreases as the number of rounds increases, and there is a strong negative correlation between the mean values of p and the round number (Pearson correlation, $r = -0.86$, $P < 0.001$). In Stage II, the mean values of p increase rapidly in the first 10 rounds. In the last 10 rounds, the mean value of p in T3 is higher than that in T1 and T2. **(B)** Time evolution of the mean values of q. In Stage I, the mean values of q decrease slowly over rounds. In Stage II, the mean values of q in T1, T2 and T3 increase over rounds. **(C)** Time evolution of the mean values of $\Delta\pi$. In Stage I, there is a strong correlation between the mean values of $\Delta\pi$ and rounds (Pearson correlation, $r = 0.93$, $P < 0.001$). In Stage II, the mean value of $\Delta\pi$ in T3 is lower than in T1 and T2. **(D)** Time evolution of mean values of π. In Stage II, the mean values of π in T3 is slightly larger than T1 and T2.

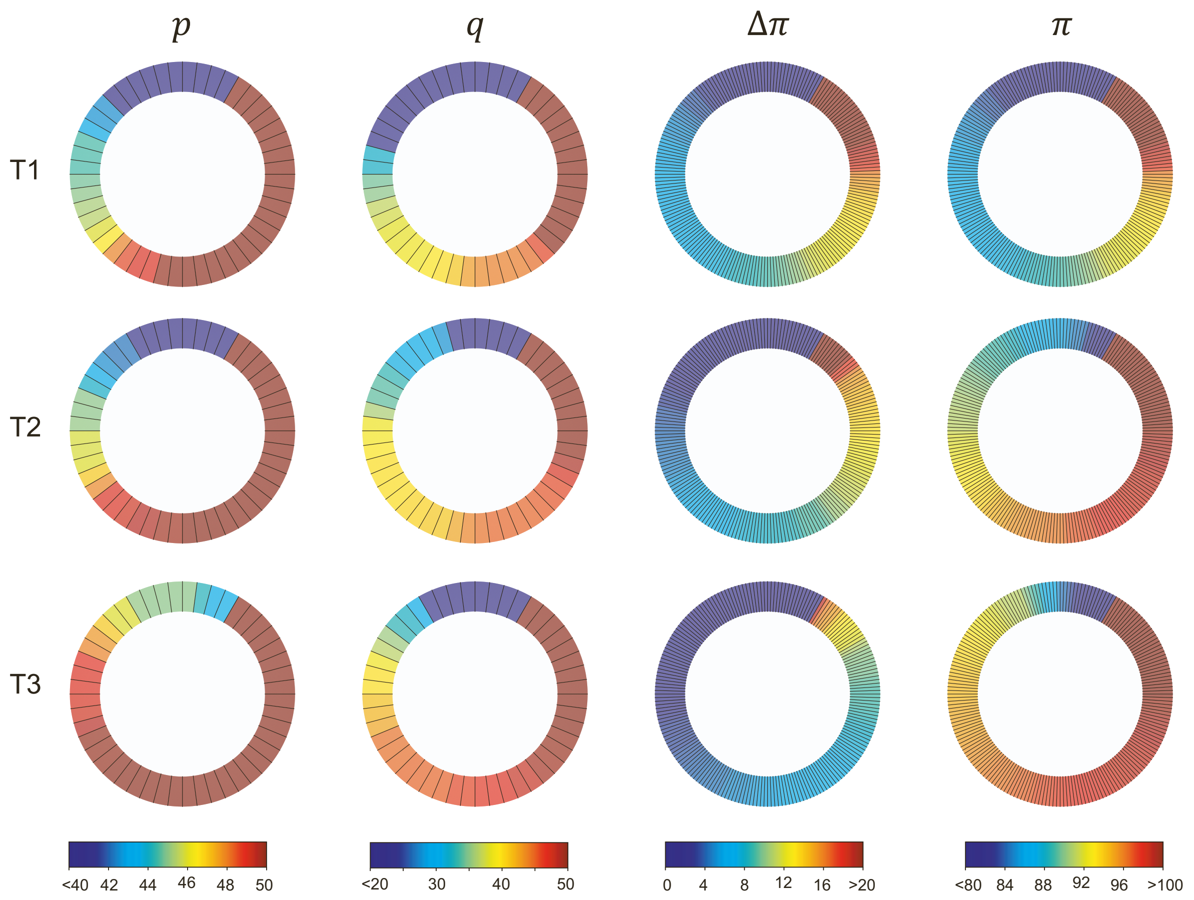

Supplementary Figure S2: Mean values of p_i, q_i, $\Delta\pi_{ij}$ and π_{ij} over the last 10 rounds of T1, T2, and T3. Each cell in the rings denotes the mean values of **(A)** p_i, **(B)** q_i, **(C)** $\Delta\pi_{ij}$ or **(C)** π_{ij} over the last 10 rounds. These values are arranged from small to large in counter-clockwise order. The four color bars represent the values of p, q, $\Delta\pi$ and π.

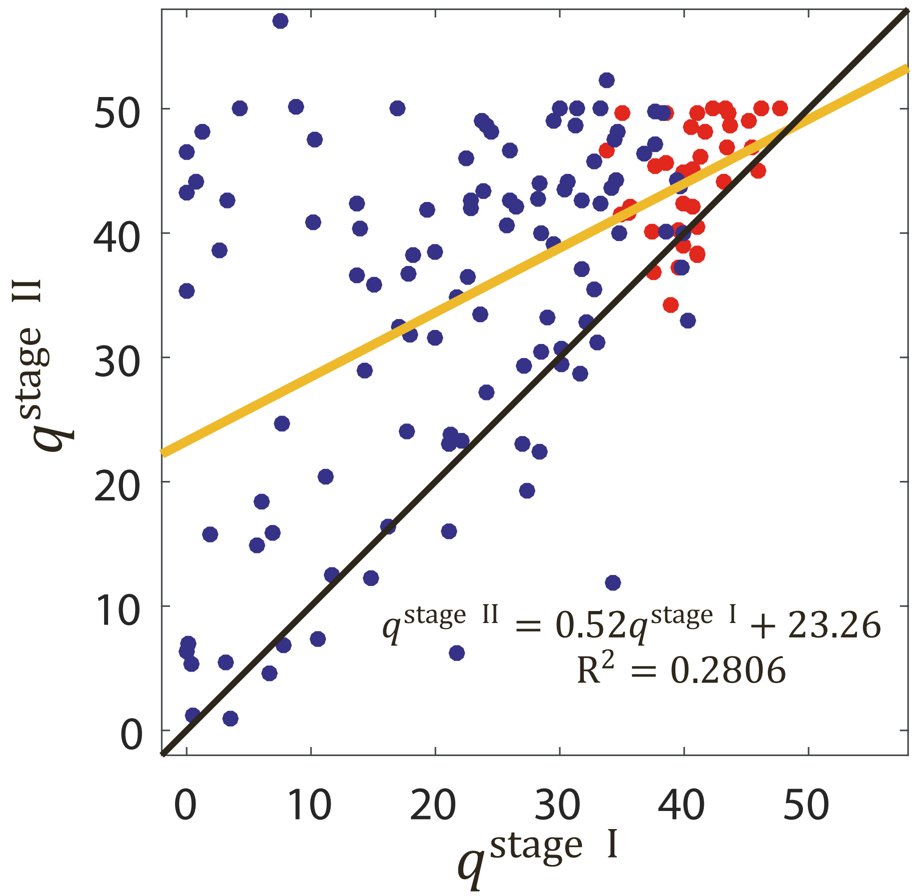

Supplementary Figure S3: Correlation of q values at individual level between stage I and stage II in T1, T2 and T3. Red and blue nodes represent leaders and other responders, respectively. The yellow line denotes the linear regression result between the two stages. Most of responders increase their q in stage II in contrast to the first stage. In particular, the leaders who have higher q in stage I also have higher q in stage II.

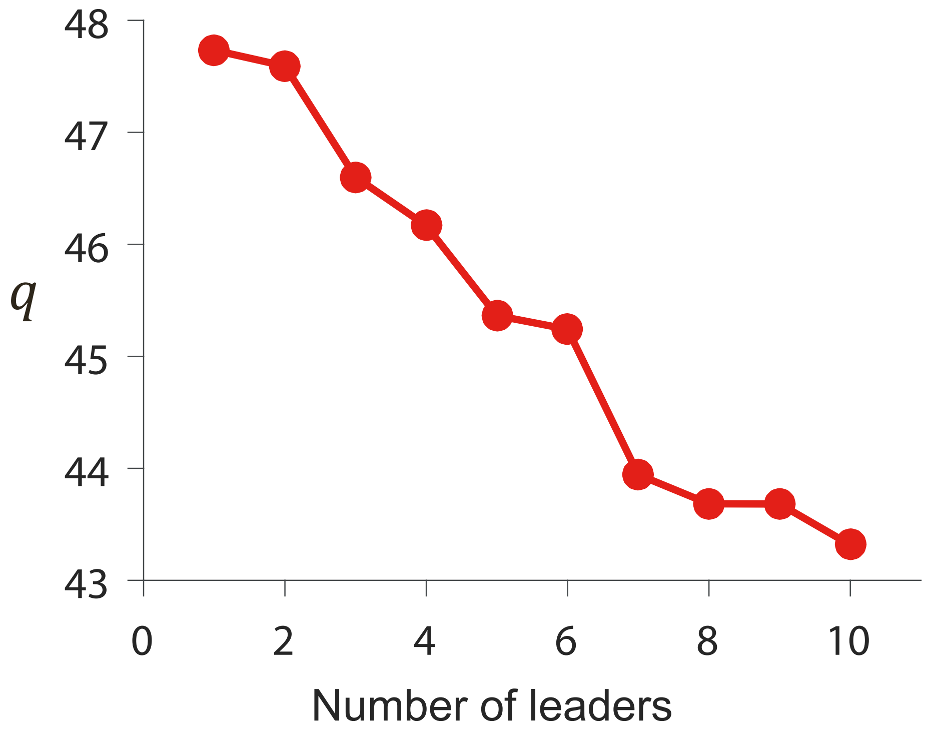

Supplementary Figure S4: The mean acceptance level of leaders as a function of their number. The responder leaders are chosen from in T1, T2 and T3 for the calculation.

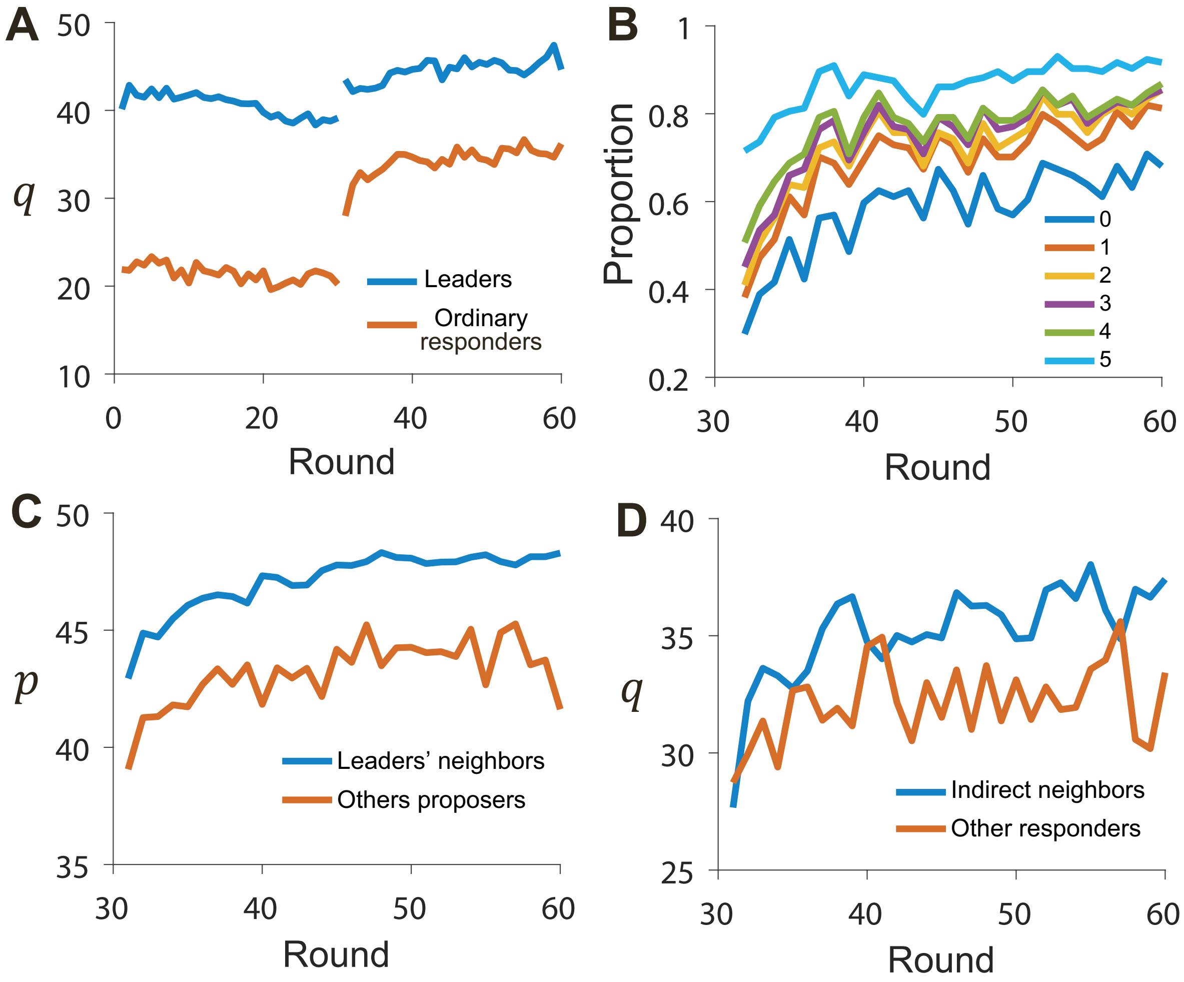

Supplementary Figure S5: Time evolution of individual behaviors and strategies in T1, T2 and T3. **(A)** Time evolution of the mean values of q for leaders and ordinary responders in stage I and II. **(B)** Time evolution of the proportion of proposers using the best-response strategy in stage II. The values in the figure legend denote the maximum distance between p and rigorous best-response strategy p_{BR}, where $0 \leq p \leq 100$. In the last 10 rounds, more than 60% of proposers exhibited rigorous best-response behaviors. **(C)** Time evolution of p for the proposers who connect with leaders (leader's neighbors) and that of the other proposers in stage II. **(D)** Time evolution of q for the responders who share proposers with leaders (indirect neighbors) and that of the other ordinary responders.

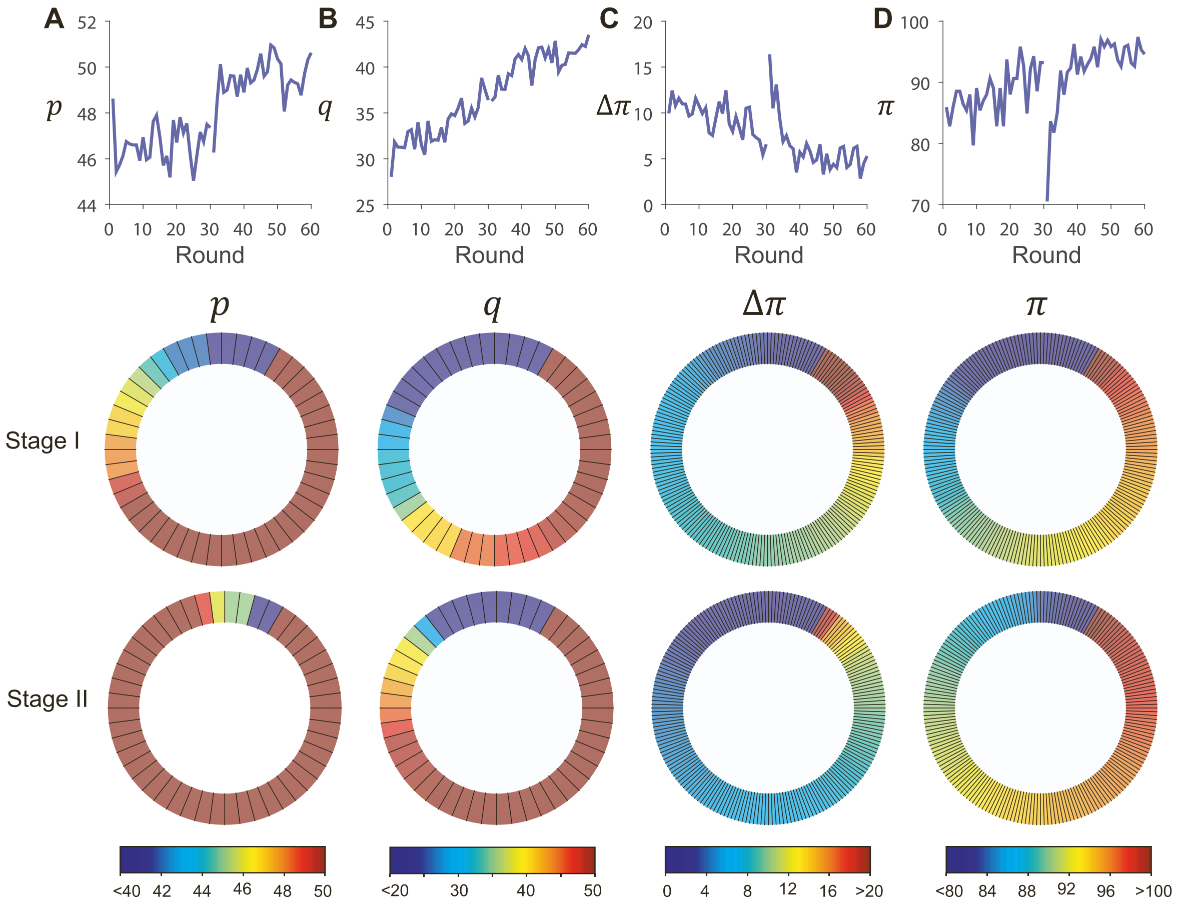

Supplementary Figure S6: Fairness and efficiency in T4. (A-D) Time evolution of p, q, $\Delta\pi$ and π. Each cell in the rings denotes the mean values of p_i, q_i, $\Delta\pi_{ij}$ and π_{ij} over the last 10 rounds of stage I or stage II. These values are arranged from small to large in counter-clockwise order. The four color bars represent the values of p, q, $\Delta\pi$ and π.

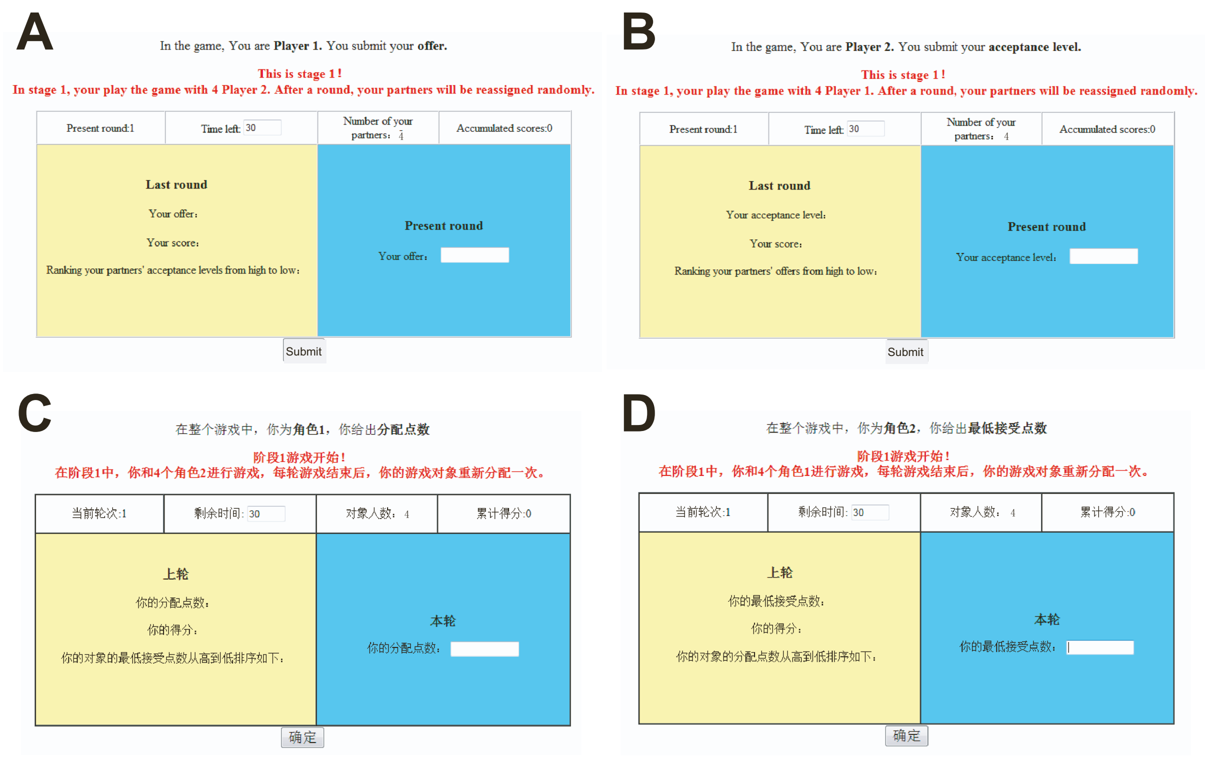

Supplementary Figure S7: Screen shots of experimental interfaces in stage I of T1, T2 and T3. (A-B) English translation of the interfaces for proposers and responders. **(C-D)** Chinese interfaces for proposers and responders. We used Chinese interfaces in the experiments. The interfaces for stage II of T1, T2, T3, and the two stages of T4 are similar.

8 Supplementary Tables

Supplementary Table S1: Mean values/standard deviation of p, q $\Delta\pi$ and π in stage II of T1, T2 and T3.

	p		q	
	All 30 rounds	Last 10 rounds	All 30 rounds	Last 10 rounds
T1	44.21/7.57	44.84/8.30	33.80/14.46	35.28/16.87
T2	45.33/4.34	46.37/5.14	37.65/11.74	38.54/11.91
T3	47.88/2.24	48.72/2.18	39.06/12.56	39.64/13.68
	$\Delta\pi$		π	
	All 30 rounds	Last 10 rounds	All 30 rounds	Last 10 rounds
T1	10.90/7.10	10.16/8.76	88.33/7.44	90.57/8.31
T2	9.45/4.71	7.55/5.78	90.47/5.95	94.01/5.62
T3	7.24/2.73	5.14/4.05	91.02/5.52	94.80/6.30

Supplementary Table S2: Simulation results of p, π, and $\Delta\pi$ across empirical bipartite networks across empirical bipartite networks with varying numbers of proposers N_p, responders N_q, average degrees of responders $\langle k \rangle_q$, and coefficient of variation CV_q of responders. Each data point represents the average of 100 simulations.

Network	N_p	N_q	$\langle k \rangle_q$	CV_q	Random/Peripheral/Central		
					p	π	$\Delta\pi$
CEO club [5]	25	15	6.33	0.78	40.84/29.98/47.68	96.55/92.98/98.12	14.90/33.55/2.99
	15	25	3.80	0.34	41.97/34.95/47.15	95.73/92.54/98.79	10.91/20.40/3.69
Elite [6]	20	24	4.13	0.76	43.06/30.65/47.68	97.00/95.59/98.77	10.07/32.29/3.05
	24	15	4.95	0.42	41.73/35.91/46.35	97.55/98.10/98.60	11.46/22.28/5.53
Baseball [7]	61	23	3.65	2.44	30.62/4.97/48.06	98.74/99.52/99.77	32.00/73.31/8.52
	23	61	1.38	0.87	35.36/5.74/49.51	98.19/99.87/98.00	12.43/40.81/10.91
Crime [8]	830	550	2.68	0.75	32.52/16.81/44.61	98.28/97.91/98.78	24.63/47.07/9.33
	550	830	1.78	1.21	37.59/20.43/47.56	97.57/95.33/97.20	18.04/43.60/5.05
SI_2016 [9]	514	1278	2.78	2.93	38.24/12.90/44.66	97.58/95.37/97.06	7.83/61.71/7.16
	1278	514	6.91	1.86	33.30/12.34/45.87	98.40/95.22/99.83	16.22/61.68/4.28
RO_2016 [9]	931	2712	3.09	2.70	38.22/19.06/42.19	98.03/92.49/98.49	6.17/68.62/6.24
	2712	931	9.00	1.93	33.75/12.62/45.59	98.23/96.02/99.87	14.74/76.39/3.72
NYC [10]	2060	2876	4.68	1.09	41.12/23.40/47.81	96.80/89.75/99.81	9.05/42.22/1.23
	2876	2060	6.54	0.97	39.46/22.63/47.23	96.92/90.96/99.83	11.64/44.18/1.87
Escorts [11]	6634	10096	3.87	1.62	37.02/15.16/46.35	97.89/85.68/99.85	8.71/56.03/1.56
	10096	6634	5.89	2.48	36.31/10.62/47.42	97.47/92.13/99.88	12.84/69.81/1.73
Mammal [12]	12234	18282	3.37	2.44	36.27/14.70/45.26	97.77/89.53/99.31	10.10/63.20/3.18
	18282	12234	5.03	2.20	34.80/11.44/46.01	97.95/93.13/99.64	14.06/71.00/3.56

Supplementary Table S3: Details of experimental conditions in T1, T2 and T3. 3 conditions of networked UG experiments are conducted: the random condition (T1), the peripheral condition (T2), and the central condition (T3). In the 'Interaction type' column, 'Well-mixed' means that subjects randomly encounter other subjects in each round, and 'Fixed' means that subjects play UG on a static bipartite network and their related players are unchanged. In the 'Network type' column, 'None' means that there is no network structure, 'Regular' denotes regular bipartite network, and 'Heterogeneous' denotes heterogeneous bipartite network. In the 'Condition' column, 'Random' means that subjects are randomly placed at a location in a regular bipartite network, and 'Peripheral' and 'Central' mean that the 6 leaders occupy nodes with degree $k = 3$ and degree $k = 7$, respectively.

Treatment	Stage I		Stage II		
	Interaction type	Network type	Interaction type	Network type	Condition
T1	Well-mixed	None	Fixed	Regular	Random
T2	Well-mixed	None	Fixed	Heterogeneous	Peripheral
T3	Well-mixed	None	Fixed	Heterogeneous	Central

9 Supplementary References

1. Ellison, G. (1993). Learning, local interaction, and coordination. Econometrica: Journal of the Econometric Society *61*, 1047–1071.

2. Brenner, T., and Vriend, N.J. (2006). On the behavior of proposers in ultimatum games. Journal of Economic Behavior & Organization *61*, 617–631.

3. Zhang, B., Cao, Z., Qin, C.Z., and Yang, X. (2018). Fashion and homophily. Operations Research *66*, 1486–1497.

4. Barabási, A.L., and Albert, R. (1999). Emergence of scaling in random networks. Science *286*, 509–512.

5. Faust, K. (1997). Centrality in affiliation networks. Social Networks *19*, 157–191.

6. Barnes, R., and Burkett, T. (2010). Structural redundancy and multiplicity in corporate networks. International Network for Social Network Analysis *30*, 4–20.

7. Krebs, V. (2008). The spread of steroids in baseball. http://orgnet.com/steroids.html. Accessed on March 26, 2025.

8. Decker, S., Kohfeld, C.W., Rosenfeld, R., and Sprague, J. (1991). St. louis homicide project: Local responses to a national problem. A report made to the community pp. 22–23.

9. Wachs, J., Fazekas, M., and Kertész, J. (2021). Corruption risk in contracting markets: a network science perspective. International Journal of Data Science and Analytics *12*, 45–60.

10. Yang, D., Zhang, D., Yu, Z., and Yu, Z. (2013). Fine-grained preference-aware location search leveraging crowdsourced digital footprints from lbsns. In Proceedings of the 2013 ACM international joint conference on Pervasive and ubiquitous computing. pp. 479–488.

11. Rocha, L.E., Liljeros, F., and Holme, P. (2011). Simulated epidemics in an empirical spatiotemporal network of 50,185 sexual contacts. PLoS Computational Biology *7*, e1001109.

12. Dallas, T.A., Aguirre, A.A., Budischak, S., Carlson, C., Ezenwa, V., Han, B., Huang, S., and Stephens, P.R. (2018). Gauging support for macroecological patterns in helminth parasites. Global Ecology and Biogeography *27*, 1437–1447.
